# Group A *Streptococcus* strains causing meningitis without distinct invasive phenotype

**DOI:** 10.1002/mbo3.1394

**Published:** 2024-01-04

**Authors:** Laura Marquardt, Federica Andreoni, Mathilde Boumasmoud, Tiziano A. Schweizer, Dorothea M. Heuberger, Elena Parietti, Sanne Hertegonne, Jana Epprecht, Dario Mattle, Anna K. Raez, Ewerton Marques‐Maggio, Reto A. Schuepbach, Barbara Hasse, Srikanth Mairpady‐Shambat, Silvio D. Brugger, Annelies S. Zinkernagel

**Affiliations:** ^1^ Departement of Infectious Diseases and Hospital Epidemiology, University Hospital Zurich University of Zurich Zurich Switzerland; ^2^ Institute for Intensive Care Medicine, University Hospital Zurich University of Zurich Zurich Switzerland; ^3^ Division of Clinical Pathology, University Hospital Zurich University of Zurich Zurich Switzerland

**Keywords:** Group A *Streptococcus*, meningitis, virulence determinants

## Abstract

Group A streptococcal (GAS; aka *Streptococcus pyogenes*) meningitis is a fulminant disease associated with high morbidity and mortality. To elucidate the mechanisms underlying the invasiveness of GAS in meningitis, we compared GAS isolates derived from five cases of meningitis to otitis and colonizing isolates. We did not observe differences in adherence to and invasion of human brain microvascular endothelial cells, virulence factors activity, or barrier disruption. Whole genome sequencing did not reveal particular invasiveness traits. Most patients previously suffered from otitis media suggesting that meningitis likely resulted from a continuous spread of the infection rather than being attributable to changes in the pathogen's virulence.

## Graphical abstract

Group A streptococcal (GAS) meningitis causes high morbidity and mortality. To elucidate the invasiveness of GAS in meningitis, we compared five GAS meningitis isolates to otitis and colonizing isolates. Genetic and virulence traits were comparable for all strains suggesting that meningitis likely resulted from a continuous spread of the infection.

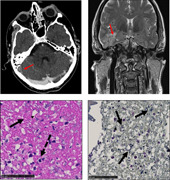

## 1 INTRODUCTION

*Streptococcus pyogenes* or group A *Streptococcus* (GAS) asymptomatically colonizes the human throat and skin. In addition to asymptomatic colonization, GAS causes mild to life‐threatening infections, such as necrotizing fasciitis and meningitis. GAS meningitis cases are rare and represent only 1%–4% of all invasive GAS cases detected across Europe and the United States (Imöhl et al., 2010; Lamagni et al., 2008; O'Loughlin et al., 2007). GAS meningitis is a fulminant disease associated with high morbidity, including neurologic sequelae, and mortality. It occurs mainly in patients with predisposing factors such as otitis, sinusitis, recent head injury or neurosurgery, the presence of a neurosurgical device, cerebrospinal fluid leak, or alcoholism (Lucas et al., 2015; van de Beek et al., 2002). This suggests extension per continuitatem from a neighboring infection such as otitis media, resulting in otogenic meningitis. However, in a third of cases, there are no predisposing factors present. The prevalence of the predisposing factors for GAS meningitis in children is acute otitis media and acute mastoiditis (2%–3% (Coker et al., 2010; Segal et al., 2005) and 11% (Laulajainen‐Hongisto et al., 2014), respectively). GAS is also responsible for 41% of acute mastoiditis and 14% of acute otitis cases that require hospitalization in children (Laulajainen‐Hongisto et al., 2015; Mansour et al., 2019) and for 19% of acute otitis and mastoiditis cases requiring hospitalization in adults (Laulajainen Hongisto et al., 2016). The most common host‐associated risk factors for invasive GAS infections in adults are crowding, diabetes, cardiac disease, cancer, and corticosteroid use (Factor et al., 2003). Other risk factors are breaching of the physical barriers of skin and mucosa by trauma or preceding viral infections such as varicella (Zangarini et al., 2023) and influenza (Oliver et al., 2019; van Kempen et al., 2023).

On the pathogen's side, GAS components such as the streptococcal virulence factors Streptolysin O (SLO), streptococcal DNases, the IL‐8 protease SpyCEP, and the M‐protein, play key roles in the invasiveness and ability of the pathogen to successfully invade and survive within the host environment (Cole et al., 2011). SLO is a pore‐forming toxin that can lead to the inhibition of phagocytosis and cell lysis of target cells (Timmer et al., 2009). The DNase sda1 was shown to degrade neutrophils extracellular traps and to prevent TLR9‐dependent recognition, leading to enhanced pathogen spread in the host (Buchanan et al., 2006; Uchiyama et al., 2012; Walker et al., 2007), while SpyCEP is involved in degradation of the cytokine IL‐8, impairing neutrophils recruitment to the site of infection (Zinkernagel et al., 2008).

The M‐protein is a GAS surface protein promoting GAS survival in the host by increasing adherence to host cells (Okada et al., 1995) escape from opsonophagocytosis (Oehmcke et al., 2010), and neutrophil activation, leading to inflammation (Herwald et al., 2004). To date, more than 200 different M‐protein types (*emm*‐types) have been described based on the sequence of the variable region of the M‐protein. Genotyping methods used for the classification of GAS strains include serotyping of the M‐protein as well as Multilocus Sequence Typing (MLST), based on the sequences of seven housekeeping genes (Enright et al., 2001). MLST is normally used to study the epidemiology and population genetics of a specific set of isolates, proving particularly useful for the detection and classification of outbreaks. Strains of *emm* type 1 (*emm*‐1) and MLST‐28 (ST28) or emm‐28 ST52 are highly prevalent among GAS meningitis isolates (Plainvert et al., 2016).

The aim of this study focused on assessing whether specific pathogen traits distinguish GAS meningitis isolates from colonizing GAS isolates or GAS‐causing otitis and whether these specific traits could be associated with disease severity. We investigated five patients suffering from GAS meningitis, analyzing both host and pathogen characteristics. On the host side, various clinical parameters were analyzed. On the pathogen side, we phenotypically and genetically compared five GAS isolates derived from the abovementioned meningitis cases with 10 GAS isolates derived either from otitis patients or asymptomatic colonization. To our knowledge, this is the first study comparing the virulence traits of meningitis isolates with otitis or colonizing ones.

## 2 MATERIAL AND METHODS

### 2.1 Patients

Five patients diagnosed between 2013 and 2017 with clinically confirmed community‐acquired GAS meningitis in Switzerland were retrospectively included in this study (Table 1). No specific surveillance program for GAS meningitis exists in Switzerland, and the cases were selected using a convenience sampling strategy. Otitis isolates were also selected using a convenience sampling strategy, while GAS colonizing isolates were selected to reflect the population present in the meningitis and otitis groups (*emm*‐1 and *emm*‐28 types).

**Table 1:** **Patient description**.

Patient ID Sex Age (y) Isolate Nr.	Associated health conditions	Clinical presentation	Complications	Site of GAS identification	Antibiotic treatment regimen	Death
**1** **F** **73** **CI1224**	Perforated otitis media Diabetes Hypothyreosis Depression	Altered vigilance and mental state Vertigo Hypothermia Hypotension Acute abdomen	Partial septic sinus transversus thrombosis Coma Irreversible hypoxic brain damage ICU involvement	CSF Blood Ear swab	3d ceftriaxone 3d amoxicillin 3d clindamycin	Yes
**2** **M** **55** **CI1271**	Otitis media Depression	Altered vigilance Meningism Slight one‐sided arm paralysis Otalgia Diarrhea Nausea	Coma Mastoiditis Cerebral abscess ICU involvement	Blood Mastoid tissue	2d ceftriaxone 1d amoxicillin Escalation to: 18d penicillin 5d metronidazole 5d clindamycin + Surgical intervention	No
**3** **F** **64** **CI1293**	Bicuspid aortic valve	Acute reduction of the general condition Meningism Right‐sided paresis of upper and lower limbs Right‐sided tongue deviation Anisocoria Petechial bleeds	Thrombocytopenia Acute kidney failure Aortitis Nosocomial Influenza infection	CSF Blood	Initially, ceftriaxone then changed to penicillin due to elevated transaminases, a total of 18d 3d gentamycin 15d steroids 10d vancomycin 10d clindamycin	No
**4** **F** **79** **CI407**	Otitis media (perforated) Coronary heart disease Pre‐diabetic metabolism	High temperatures Vertigo Altered mental state and vigilance	Tegmen tympani penetration Mastoiditis Labyrinthitis Coma Long‐term one‐sided deafness ICU involvement	Mastoid tissue	Initially 1d ceftriaxone, then escalation to meropenem for 2d, then back to ceftriaxone for 20 days Initially 2d acyclovir 1d amoxicillin 4d mephamesone + Surgical intervention	No
**5** **M** **42** **CI543**	Otitis media Bullous Myringitis with insertion of tympanostomy tube	One day after the insertion of the tympanostomy tube acute reduction of vigilance Altered mental state and meningism	Mastoiditis Subdural empyema Cerebritis Ventrikulitis Coma ICU involvement	Blood CSF Mastoid tissue	9d ceftriaxone, then escalation to 18d ceftazidime and de‐escalation back to ceftriaxone for another 35d 2d amoxicillin + clavulanic acid 2d acyclovir 2d metronidazole 2d, pause 3d, then another 2d 9d intrathecal gentamycin 9d intrathecal vancomycin + Surgical intervention	No

*Note*: Clinical findings, complications, and treatment regimens of the patients included in this study.

Abbreviations: CSF, cerebrospinal fluid; d, day; ICU, intensive care unit.

### 2.2 Bacterial strains and cell lines

All meningitis, otitis, and colonizing GAS clinical isolates are listed in Supporting Information: Table S1 and were cultured in THY (Todd–Hewitt broth [BD] plus 2% yeast extract [Oxoid]) at 37°C. Immortalized human brain microvascular endothelial cells (HBMECs) (Nizet et al., 1997) were cultured in Roswell Park Memorial Institute (RPMI) medium supplemented with 10% fetal bovine serum (FBS) and l‐glutamine unless specified otherwise.

### 2.3 Adherence and invasion assays

Adherence and invasion assays were performed as previously described, with minor modifications (Andreoni et al., 2014). HBMECs were seeded in 24 well plates to 2.2 × 10^4^ cells/well in RPMI medium supplemented with 10% FBS and l‐glutamine and incubated at 37°C in the presence of 5% CO_2_ for 48 h. Overnight (ON) cultures of the GAS strains were grown at 37°C in a static incubator in 5 mL THY. Bacterial cultures were diluted 1:10 in fresh THY medium and grown to an optical density at 600 nm (OD_600_) of 0.4, washed once with phosphate‐buffered saline (PBS), and resuspended in infection medium (RPMI+l‐glutamine+0.4% bovine serum albumin). Before infection, HBMECs were washed once with PBS and bacteria, resuspended in 500 μL of infection medium, and were added to reach a multiplicity of infection (MOI) of 1 or 10 for adherence and invasion assays, respectively. The plates were spun down at 1200*g* for 5 min and subsequently incubated at 37°C in the presence of 5% CO_2_. To assess adherence, infected HBMECs were incubated for 30 min, washed six times with PBS to remove unbound bacteria, and subsequently lysed using 500 μL of deionized sterile water (dH_2_O). To assess invasion, infected HBMECs were incubated for 2 h and washed three times with PBS to remove excess bacteria. 500 μL of infection medium supplemented with penicillin and gentamycin, at a final concentration of 10 μg/mL and 100 μg/mL, respectively, were added to each well to kill extracellular bacteria. The plates were then incubated for 2 h at 37°C in the presence of 5% CO_2_, and the cells were subsequently lysed with 500 μL of dH_2_O after three washes with PBS. Ten‐fold serial dilution of the cell lysates was plated on THY plates to enumerate bacterial colonies after ON incubation at 37°C. The percentage of adherence and invasion was calculated relative to the initial inoculum.

### 2.4 Viability assay

Cell viability was assessed as follows. HBMECs and bacteria were prepared for adherence and invasion assays, as described above. After 30 min of infection followed by six washes with PBS or after the 2 h incubation with antibiotics followed by three washes with PBS, HBMECs were harvested using 100 µL of trypsin (Gibco), washed, and resuspended in 500 µL PBS. Three wells/conditions were pooled, and the experiment was carried out in duplicate.

250 µL of the cell suspension were transferred to the wells of a conical 96‐well plate, spun down at 470 g for 5 min, washed with Annexin V binding buffer (Thermo Fisher), and spun down at 470 g for 5 min. Dead cells were stained using 40 µL/well of a cocktail of Annexin V‐FITC (#640906; Biolegend, 1:50 dilution) and 7 AAD (#420404; Biolegend 1:25 dilution) diluted in Annexin V binding buffer, for the detection of apoptotic and late apoptotic cells. The cells were incubated for 20 min at room temperature, 160 µL of Annexin V binding buffer was added, and data were acquired using the AttuneNxT flow cytometer (Thermo Fisher).

### 2.5 Virulence factors activity

The activity of the virulence factors SLO, streptococcal DNases, and SpyCEP was assessed in the supernatants of exponentially growing GAS as previously described (Andreoni et al., 2017). ON cultures of the various strains were statically grown at 37°C in THY medium. Bacterial cultures were diluted 1:10 in fresh THY medium and grown to an optical density at 600 nm (OD_600_) of OD 0.4. After centrifugation, the supernatants were removed and filter sterilized using 0.22 µm filtration membranes.

SLO activity was assessed as follows. 4 mM DTT and 0.0004% Trypan‐blue were added to the supernatants, the samples were incubated for 10 min at room temperature (RT), and twofold dilutions were made in PBS in a 96‐well plate. PBS and dH_2_O alone were used respectively as negative and positive controls. Erythrocytes were diluted to 2% v/v in PBS and added to the diluted supernatants (one part erythrocytes and four parts supernatant) or PBS or water for no lysis and full lysis controls, respectively. Samples were incubated at 37°C for 30 min and spun down at 3000*g* for 5 min. The OD_541_ of the supernatants, directly proportional to the activity of SLO, was used as a readout.

Streptococcal DNase activity was assessed as follows. 5 μL of bacterial supernatant or 5 μL of THY (negative control) were mixed with 5 μL of 10X DNase buffer (50 mM CaCl_2_, 500 mM Tris, pH 7.9) and 5 μL of calf thymus DNA (1 mg/mL, Sigma) in a total volume of 50 μL and incubated at 37°C for 5 min. The reaction was stopped by adding 8 μL of 0.5 M EDTA, and the samples were loaded on a 0.8% agarose gel to assess DNA degradation. Streptococcal DNase activity was evaluated with a score corresponding to no activity (0 = no degradation of calf thymus DNA), intermediate activity (1 = partial degradation of calf thymus DNA), and high activity (2 = complete degradation of calf thymus DNA).

SpyCEP activity was assessed by measuring the degradation of the cytokine IL‐8. Bacterial supernatants were incubated with 1 ng/μL IL‐8 for 16 h at 37°C after which an IL‐8 ELISA was carried out according to the manufacturer's instruction (R&D). THY was used as a negative control. SpyCEP activity is depicted as the percentage of IL‐8 cleavage compared to the negative control (medium only).

### 2.6 Electric cell‐substrate impedance sensing (ECIS®) assay

ECIS® was performed with the ECIS® Z‐Theta instrument (Applied Biophysics) to monitor the effects of the GAS strains on human brain microvascular endothelial cells (HBMECs) (Nizet et al., 1997) barrier integrity. Before seeding, the wells of the arrays (ECIS 8W10E+; IBIDI) were washed twice with sterile, nuclease‐free dH_2_O, coated with 5 µg/mL collagen IV for 1 h at room temperature (RT), washed three times with dH_2_O and incubated with 10 mM l‐cysteine for 2 h at RT. The wells were washed two times with dH_2_O before 200 µL of culture medium was added and the assay started (37°C, 5% CO_2_). After 30 min, 200 µL culture medium containing 10^5^ HBMECs was seeded per well. After 22 h incubation 50 µL of medium were removed, and 2 h later HBMECs were infected with log phase GAS strains resuspended in DMEM+10%FCS at an MOI of 10. DMEM+10%FCS alone was used as a control. After 1.5 h penicillin (10 µg/mL) and gentamycin (100 µg/mL) were added. Barrier integrity was monitored at 4000 Hz from seeding to 70 h postinfection. Resistance data were normalized to the time of infection.

### 2.7 Bacterial genomics

Whole genome sequencing and genomic data analysis were performed as previously described (Räz et al., 2023). Briefly, from 150 bp paired‐end reads, de novo assemblies were generated with SPAdes v.3.10 (Räz et al., 2023) and annotated with Prokka v.1.13 (Seemann, 2014). A pangenome was constructed with Roary v.3.12 (Page et al., 2015), and the resulting core‐gene alignment was used to build a maximum likelihood phylogenetic tree with Fasttree v.2.1.10 (Price et al., 2009). *Emm*‐typing was performed in silico by querying assemblies against the curated CDC database (CDC CfDCaP, 2021; Control CoD, 2023) with blastn v.2.9.0. Virulence genes were identified by querying the assemblies against the full VFDB database, that is, VFDB_setB_nt.fasta, downloaded on the 04.08.23, (VFDB, 2023) using ABRIcate v.0.5 (minimum identity and coverage: 85%). ENA accession number: PRJEB57816.

### 2.8 Statistics

Three biological replicates were carried out for each strain, except for HBMECs viability and ECIS assessment where two biological replicates were carried out for each strain. The average of the biological replicates for each strain is represented on the graphs as a single data point. The average of these values represents the final value depicted on the graphs. Error bars represent standard deviations. The Mann–Whitney *U* test was used to assess statistical significance for all assays apart from HBMECs viability data that were analyzed using the Kruskal–Wallis test.

## 3 RESULTS

### 3.1 Cases description

**Figure 1:**
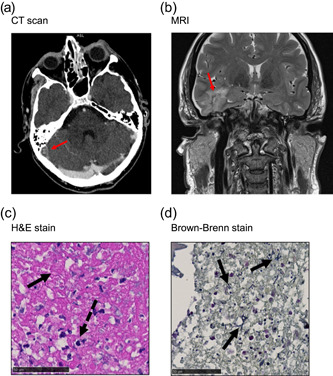
Patient scans and histology results. (a) CT‐scan: Septic thrombosis with mastoiditis and otitis media on the right side. The red arrow indicates thrombosis. (b) MRI‐Scan: T2 sequence showing penetration of lower temporal lobe with abscess formation. The red arrow indicates an abscess. (c) HE Staining of mastoid tissue: the black arrow indicates necrotic mastoid tissue, and the dashed black arrow indicates mixed neutrocytic and lymphocytic infiltration, foam cells, and cholesterol crystals (not depicted) indicating chronic inflammation. (d) Brown–Brenn staining of mastoid tissue: The arrows indicate the accumulation of extracellular bacteria in chains. CT, computed tomography; H&E, hematoxylin and eosin; MRI, magnetic resonance imaging.

Five GAS meningitis cases occurring between 2013 and 2017 in Switzerland were included in this study and the corresponding GAS isolates were retrospectively retrieved for analysis. The median age of the patients was 64 years (42–79 years) and 60% (*n* = 3) were female. The mortality rate was 20% (*n* = 1), while two out of six patients (33%) experienced neurological sequelae (Patients 3 and 4), which included slight hearing loss, vertigo, and slight paresis of the limbs. The median duration of the hospital stay was 17 days (3–29 days) and four patients needed intensive care treatment with endotracheal intubation. Relevant clinical and laboratory findings are depicted in Table 1. Mastoiditis was the underlying diagnosis in three cases. During the course of the disease, one patient had septic thrombosis, and one developed a brain abscess (Figure 1a,b). Surgical treatment such as antrotomy, mastoidectomy, or burr‐hole trepanation was required in three patients. In Patient 4, histological analysis from the surgically removed mastoid tissue showed necrotic tissue and individual mucosal fragments covered by simple cuboid epithelium with severe acute and chronic inflammation and showing extracellular accumulation of bacterial cells (Figure 1c,d).

### 3.2 Bacterial virulence

**Figure 2:**
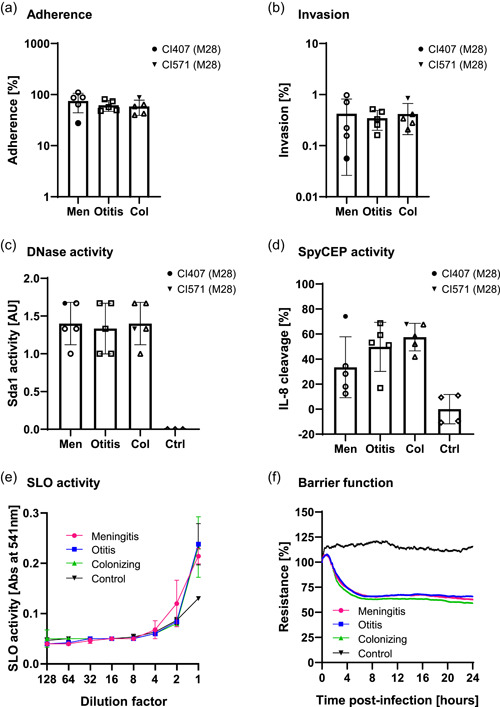
Bacterial strains virulence determinants. (a) Adherence to and (b) invasion of human brain vascular endothelial cells (HBMECs) were assessed after 30 min (MOI 1) or 2 h (MOI 10) of infection, respectively. Adherence and invasion percentages were calculated based on the initial bacterial inoculum used to infect HBMECs. Bacterial adherence above 100% occurred in one case and could be explained by increased initial bacterial growth or by disruption of long bacterial chains or clumps due to sheer pipetting forces during the eukaryotic cell lysis step. Bacterial virulence factors activity of (c) Streptococcal DNases, (d) the IL‐8 protease SpyCEP, and (e) the pore‐forming toxin SLO were assessed in supernatants of exponentially growing GAS. (f) HBMECs barrier function disruption was carried out with the ECIS® Z‐Theta instrument (Applied Biophysics), measuring the impedance generated by the cell barrier in the presence or absence of bacteria. Control experiments were carried out using medium only. (a–d) The average of the biological replicates for each strain is depicted as a single data point on the graphs so that each data point represents a single strain. The two strains indicated in the figure legend with a black symbol (CI407 and CI571) are the only strains of *emm*‐type 28 (M28), as opposed to all other strains that are *emm*‐type 1 (M1). (a–e) Three biological replicates were carried out for each strain. The average values for strains from a single group of isolates (meningitis, otitis, or colonizing) are depicted in the graphs and the error bars represent the standard deviation. (f) Two biological replicates were carried out for each strain and the average of the strains from to the same group is depicted on the graph. Control = medium only. No significant differences in adherence, invasion, virulence factors activity, or barrier disruption were observed among the different groups of isolates. A significant difference was found between the respective controls and meningitis, otitis, or colonizing isolates for DNase (*p* = 0.0159, 0.0159, and 0.0159, respectively) and SpyCEP (*p* = 0.0179, 0.0357, and 0.0179, respectively) activity. Statistical significance was assessed using the Mann–Whitney test. Col, colonizing; Ctrl, control; Men, meningitis.

The assessment of adherence to and invasion of HBMECs, the activity of the streptococcal virulence factors DNases, SLO, and SpyCEP as well as barrier integrity of HBMECs upon GAS challenge was carried out on meningitis, otitis, and colonizing clinical isolates. No significant difference between adherence and invasion behavior to HBMECs upon infection with meningitis, otitis, or colonizing GAS isolates was found (Figure 2a,b) and the toxicity of the isolates toward HBMECs during infection did not vary according to their sampling location (Supporting Information: Figure S1A and S1B). Furthermore, no differences in virulence factors activity or the isolate's potential to disrupt the barrier formed by HBMECs among the three groups were observed (Figure 2c–f). A trend, albeit not significant, towards reduced SpyCEP activity was observed in the meningitis group (Figure 2d).

### 3.3 Genotyping

Typing of meningitis and otitis isolates showed a high prevalence of emm‐1/ST28 (*n* = 4/5 and *n* = 5/5, respectively, Supporting Information: Table S1). Since one meningitis clinical isolate belonged to the emm‐28/ST52, we chose to include four emm‐1/ST28 and one emm‐28/ST52 in the colonizing isolates group. Only one meningitis clinical isolate belonged to the *emm*‐28/ST52, and one colonizing isolate to the *emm*‐28/ST458. A core genome phylogeny revealed that, within the M1 clade, the meningitis isolates did not form a monophyletic group (Supporting Information: Figure S2A and S2B). The presence of genes encoding virulence factors was analyzed and this showed no specific pattern for meningitis isolates when compared to otitis or colonizing ones (Supporting Information: Figure S2C). We additionally checked for the presence of mutations in the GAS CovRS regulatory genes that may lead to upregulated virulence factors activity (Supporting Information: Table S2) (Hollands et al., 2010). No mutations were found in CovR, while we found CovS mutations in two of the meningitis isolates (CI1224, *emm*‐1 and CI407, *emm*‐28) and in one of the colonizing ones (CI571, *emm*‐28). An in‐frame deletion of the proline in position 16 was found in CI1224. CI407 presented a deletion of the first 46 amino acids as well as non‐synonymous mutations in position 47 (Leu‐Met), 226 (Glu‐Gly) and 332 (Val‐Glu). Isolate CI571 had the same non‐synonymous mutations as CI407 in position 226 (Glu‐Gly) and 332 (Val‐Glu), common to the emm‐28 lineage (Green et al., 2005). However, these mutations did not result in a reduced CovS function or increased virulence factors activity.

## 4 DISCUSSION

We found no particular phenotypic or genotypic characteristics in the five GAS isolates causing meningitis as compared to otitis or colonizing isolates. The only common denominator was preceding otitis and mastoiditis on the host's side. GAS meningitis is an extremely rare condition, representing only 0.2%–0.9% of cases of bacterial meningitis in the United States and Brazil (Randhawa et al., 2018; Santos et al., 2009; Schlech, 1985) and 1%–4% of invasive GAS cases in Europe and the United States (Imöhl et al., 2010; Lamagni et al., 2008; O'Loughlin et al., 2007). A report from Switzerland described nine cases of GAS meningitis registered between 1983 and 1999 across all major Swiss hospitals, again emphasizing the rarity of this disease (Sommer et al., 1999). A recent report showed an increase in the number of GAS meningitis cases in the Netherlands in 2022–2023, compared with the previous 40 years, with emm‐1 being the most represented M‐protein variant (van der Putten et al., 2023).

Meningitis occurs either by hematogenous spread or by direct spread of bacteria from a neighboring infection. Hematogenous spread normally occurs when colonizing bacteria breach the nasopharyngeal mucosa, reach the blood (Hersi & Kondamudi, 2021), and subsequently cross the blood–brain barrier. It was suggested that the spread from the upper respiratory tract mucosa may be due to an altered balance in the host–microbe population, possibly favoring the GAS number increase and subsequent spread (van de Beek et al., 2002). This interesting hypothesis was, however, never tested and remains a speculation. On the other hand, direct spread to the brain can also occur per continuitatem from infected adjacent anatomic structures, such as the ear or the mastoid bone (Hersi & Kondamudi, 2021).

We searched for common traits in the clinical presentation of our patients and characteristics shared by the meningitis clinical isolates, as opposed to otitis and colonizing ones, to find possible explanations for the spreading of GAS to the brain. In our patients' cohort, the presence of mastoiditis in four patients and thrombosis in one supports the idea of direct spread through bone erosion from neighboring oto‐mastoiditis and osteo‐thrombophlebitis. Moreover, otitis, a condition often associated with GAS meningitis (van de Beek et al., 2002) was present in the majority of cases suggesting continuous spread as a likely cause of infection. In contrast, hematogenous spread was less common and a plausible cause of infection for Patient 3 only. There is no way to determine whether the presence of bacteria in the blood in Patients 1, 2, and 5 occurred before or after spread to the central nervous system. However, given the infection focus and the evidence pointing to continuous spread from the ear and mastoid bone as one of the main causes of GAS meningitis, we postulate that hematogenous spread, In the case of Patients 1, 2, and 5, was not the cause of meningitis.

The presence of a local focus on infection may explain the high surgery rate in our GAS meningitis cohort, which has been reported previously. In a nationwide cohort study in the Netherlands, 46% of GAS meningitis patients required surgical intervention, while this remains an absolute rarity only described in case reports for the more common meningitis pathogens (Pneumococcus, Meningococcus) (Lucas et al., 2015; Perin et al., 2008).

On the pathogen side, no common features explaining bacterial spread to the brain were found among the meningitis isolates, as compared to otitis and colonizing ones. This points to host rather than pathogen factors playing a role in facilitating bacterial invasion, as previously described for *S. aureus* (Räz et al., 2023 *)*. Adherence and invasion assays towards HBMECs confirmed the absence of a particular tropism towards brain microvascular tissue for the meningitis isolates, excluding an advantage in crossing or damaging the blood‐brain barrier, as compared to otitis and colonizing ones, in the tested settings. Moreover, virulence factor activity did not significantly vary among the three groups of isolates. The *emm*‐type of the strains did not generally seem to influence adherence, invasion, or virulence factors activity, although the number of strains and *emm*‐types tested is too little to be able to make a conclusive statement. A trend towards decreased SpyCEp activity was observed in the meningitis group with the M28 strain CI407 acting as an outlier and presenting a level of activity remarkably higher than the other meningitis isolates. CI407 was characterized by a pronounced deletion of the first portion of the CovS protein, a key regulator of virulence factors activity in GAS, as well as by the presence of three nonsynonymous mutations (Supporting Information: Table S2), as compared to the sequence of the CovS protein of GAS M1T1 5448 (Hollands et al., 2010). Normally a complete loss of CovS function, as for the GAS M1T1 5448 AP strain, would lead to a complete IL‐8 degradation leading to 100% cleavage (Andreoni et al., 2017), which we did not observe. SLO and DNase activities were also identical to the nonmutated strains. A partial loss of CovS functionality could be postulated in this case (Tatsuno et al., 2013), although the mutations in CI407 mainly affect the N‐terminal portion of the CovS protein where no functional domains are present (Mayfield et al., 2014; Walker et al., 2007). Further investigations involving a larger group of clinical isolates, including different *emm*‐types, are needed to address the question of whether a decreased IL‐8 degradation potential might facilitate invasion of the blood‐brain barrier. Isolates from the *emm*‐1 and *emm*‐28 lineages represented both in the meningitis and colonizing groups, behaved very similarly in terms of virulence factors activity.

In accordance with previous studies stating an association of *emm*‐1/ST28 with GAS meningitis (Plainvert et al., 2016) 4/5 meningitis isolates belonged to the *emm*‐1/ST28 lineage. The fifth isolate belonged to the *emm*‐28 lineage, which is another prevalent *emm*‐type previously described in GAS meningitis (Plainvert et al., 2016) and, together with *emm*‐1, among the most representative *emm*‐types for invasive GAS infections across Europe and North America (Gherardi et al., 2018). All otitis and colonizing isolates belonged either to the *emm*‐1 or the *emm*‐28 lineages as well. A prevalence of the emm‐1 linage in acute otitis media in children (around 30%) was previously reported (Imöhl et al., 2021). The serotype of colonizing isolates was, however, specifically selected to reflect the serotypes present in the meningitis and otitis groups. It cannot be excluded that the overrepresentation of *emm*‐1 serotypes in meningitis and otitis might reflect the global success and dominance of *emm*‐1 strains causing GAS‐invasive infections (Gherardi et al., 2018). The presence or absence of GAS virulence determinants did not reveal a specific pattern for meningitis isolates when compared to otitis and colonizing ones.

GAS meningitis occurs very rarely, leading to the availability of a small number of cases that could be included in this study. Nevertheless, despite the small sample size, our data suggest that the most common pathophysiological cause in this patients' cohort was local spread from a site of bacterial infection in neighboring tissues, with hematogenous spread a possible explanation in one case.

In the absence of distinct virulence determinants characterizing the meningitis isolates, we conclude that, in this particular cohort, host factors such as breach of the mucosa or local infections play a preponderant role in the transition from colonization to invasion. These results call for future studies, including a higher number of cases and clinical isolates and a more comprehensive phenotypic and genotypic investigation to support our conclusion.

## AUTHOR CONTRIBUTIONS

**Laura Marquardt**: Investigation; writing—original draft; data curation. **Federica Andreoni**: Investigation; writing—original draft; methodology; validation; data curation; supervision; conceptualization; writing—review & editing. **Mathilde Boumasmoud**: Investigation; writing—original draft; validation; methodology; data curation; software; writing—review & editing. **Tiziano A. Schweizer**: Investigation; data curation; methodology. **Dorothea M. Heuberger**: Investigation; data curation; methodology. **Elena Parietti**: Investigation; data curation. **Sanne Hertegonne**: Investigation; data curation. **Jana Epprecht**: Investigation; writing—original draft. **Dario Mattle**: Investigation. **Anna K Raez**: Investigation. **Ewerton Marques‐Maggio**: Visualization; investigation. **Reto A. Schuepbach**: Funding acquisition. **Barbara Hasse**: Funding acquisition. **Srikanth Mairpady‐Shambat**: Conceptualization; funding acquisition; writing—review & editing; project administration; supervision; methodology; validation. **Silvio D. Brugger**: Conceptualization; funding acquisition; writing—review & editing; project administration; supervision. **Annelies S Zinkernagel**: Project administration; conceptualization; funding acquisition; writing—review & editing; supervision.

## CONFLICT OF INTEREST STATEMENT

The authors declare no conflict of interest.

## ETHICS STATEMENT

This study was approved by the regional Committee for Medical Research Ethics (BASEC‐ID 2016‐00145 and 2017‐02225).

## Supporting information

Supporting information.

Supporting information.

Supporting information.

## Data Availability

The data that support the findings of this study are openly available in European nucleotide archive at https://www.ebi.ac.uk/ena/browser/home, reference number PRJEB57816.
