# Supplementary figures and images for: Group A Streptococcus strains causing meningitis without distinct invasive phenotype
Source: Microbiologyopen. 2024 Jan 4;13(1):e1394. doi: 10.1002/mbo3.1394 (PMC10765256; doi:10.1002/mbo3.1394)

**A****Adherence: HBMECs viability**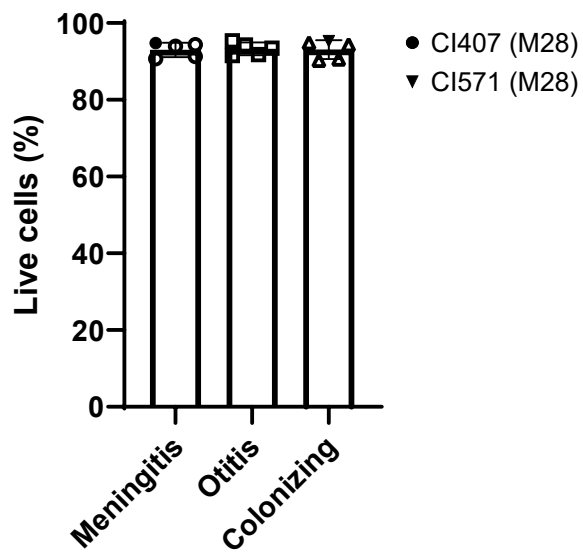**B****Invasion: HBMECs viability**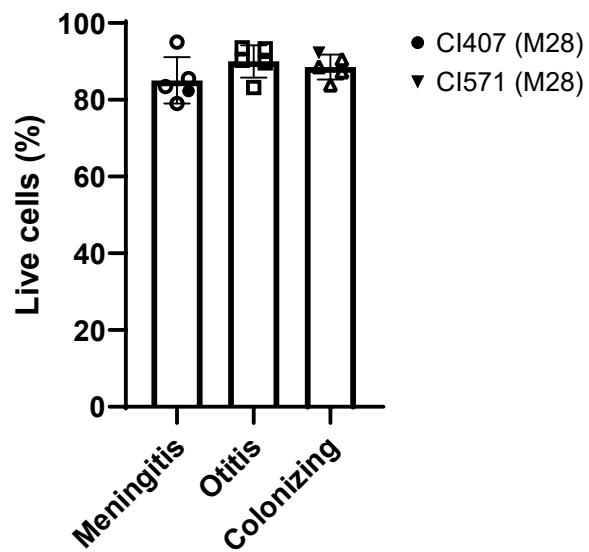

Supplement: Supplementary file 1 — Supporting information. [file MBO3-13-e1394-s002.pdf]

## Phylogenetic tree

**A**

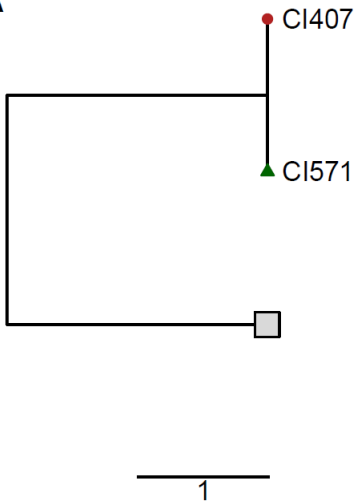

**B**

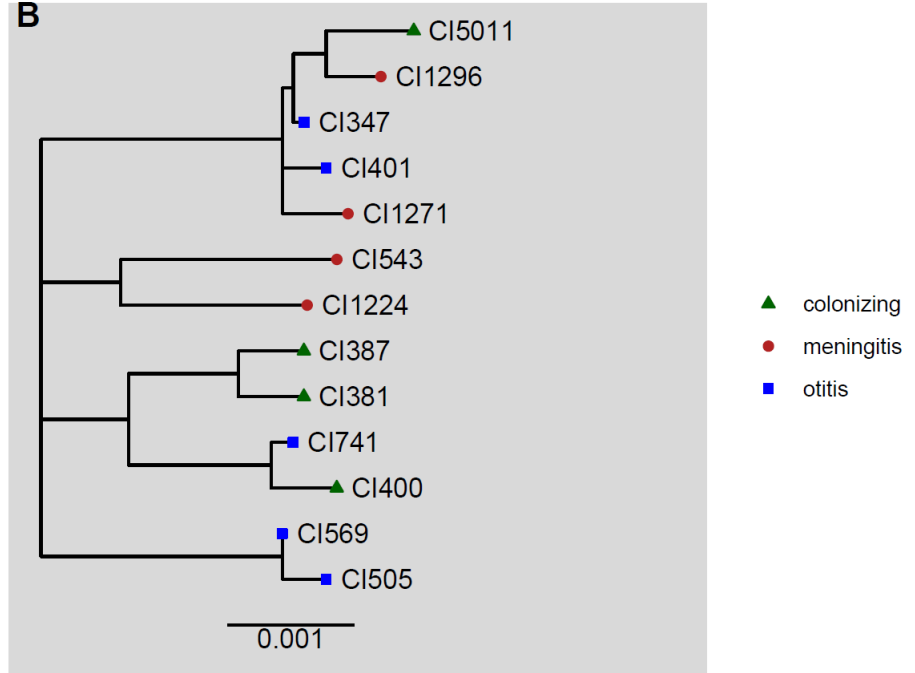

## Genetic determinants

**C**

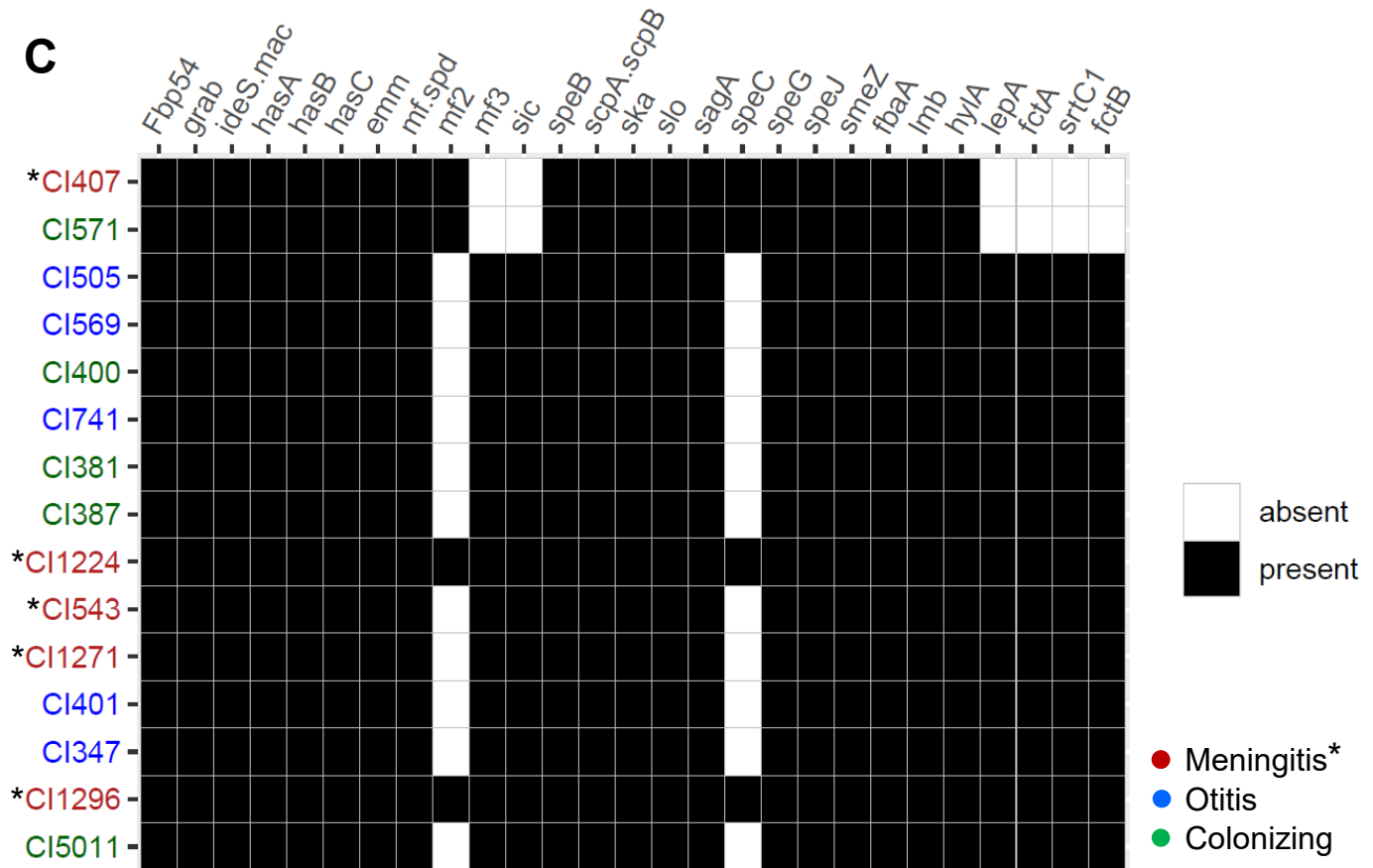

Supplement: Supplementary file 2 — Supporting information. [file MBO3-13-e1394-s001.pdf]
